# Peer Review for “Checklist Approach to Developing and Implementing AI in Clinical Settings: Instrument Development Study”

**DOI:** 10.2196/70058

**Published:** 2025-02-20

**Authors:** Saima Zaki

**Affiliations:** 1 Department of Physiotherapy, School of Allied Health Sciences, Sharda University, Plot No. 32-34, Knowledge Park III, Greater Noida, India, 91 120-457-0000

**Keywords:** artificial intelligence, machine learning, algorithm, model, analytics, AI deployment, human-AI interaction, AI integration, checklist, clinical workflow, clinical setting, literature review

*This is the peer-review report for “Checklist Approach to Developing and Implementing AI in Clinical Settings: Instrument Development Study.”*

## Round 1 Review

### General Comments

This paper [1] presents the Clinical Artificial Intelligence (AI) Sociotechnical Framework (CASoF), a checklist intended to support the development and implementation of AI systems in health care settings. The framework is built on a comprehensive literature review and a modified Delphi study involving health care professionals globally. The manuscript addresses a significant gap in the integration of AI by emphasizing the importance of sociotechnical considerations alongside technical aspects.

### Specific Comments

#### Major Comments

1. Clarity and structure: The manuscript could benefit from clearer explanations, particularly in the methodology section. The description of the Delphi study and literature synthesis is dense and may be difficult for readers to follow. Consider breaking down complex sentences and using more straightforward language.

2. Methodological rigor: The manuscript lacks details on the selection process for Delphi panelists and the exact methods used for data analysis. Transparency in these areas would significantly strengthen the paper. Additionally, clarify how the Delphi method was modified and the rationale behind these modifications.

3. Literature review and contextualization: The discussion section could benefit from a more critical comparison between the CASoF and existing frameworks. While the manuscript mentions other frameworks, it does not fully explore their limitations or how the CASoF overcomes these challenges. Expanding this discussion would provide a stronger justification for the CASoF’s novelty and utility.

4. Checklist practicality: While the checklist is comprehensive, its length and complexity may hinder practical adoption. Consider providing a condensed version for quick reference and include examples of how the checklist can be applied in real-world scenarios.

5. Ethical considerations and bias mitigation: The manuscript discusses ethical considerations but lacks specific strategies for addressing these issues within the CASoF. Expanding this discussion would enhance the manuscript’s comprehensiveness.

#### Minor Comments

6. Typographical and grammatical errors: There are minor typographical and grammatical errors throughout the manuscript that should be corrected. For instance, phrases like “revised and edited” could be simplified to “revised” for conciseness.

7. Tables and figures formatting: The tables summarizing the Delphi study results are helpful but could be more effectively formatted. Using shading or color coding to distinguish between different stages or domains would improve clarity and ease of interpretation.

8. Recent references: Some references in the manuscript are relatively old, which is less ideal for a rapidly evolving field like AI. Where possible, the manuscript should incorporate more recent literature to support its claims and demonstrate the ongoing relevance of the topic.

## Abbreviations

AI: artificial intelligence
CASoF: Clinical Artificial Intelligence Sociotechnical Framework
